# Food Insecurity and Primary School Girl Students' Intelligence Quotients: A Case-Control Study

**Published:** 2018-04-15

**Authors:** Ahmad Reza Dorosty Motlagh, Peivasteh Safarpour, Milad Daneshi Maskooni, Mostafa Hosseini, Farzaneh Ranjbar Noshari

**Affiliations:** ^1^Department of Community Nutrition, School of Nutritional Sciences and Dietetics, Tehran University of Medical Sciences, Tehran, Iran; ^2^Department of Nutrition, School of Public Health, Iran University of Medical Sciences, Tehran, Iran; ^3^Department of Epidemiology and Biostatistics, School of Public Health and Institute of Public Health Research, Tehran University of Medical Sciences, Tehran, Iran; ^4^Department of Payamnoor Psychology, Rezvanshahr University, Guilan, Iran

**Keywords:** Food Insecurity, Intelligence Quotient, Primary School, Girl, Iran

## Abstract

**Background:** Food insecurity (FI) refers to the lack of sufficient and safe availability of the food. Accumulating studies have suggested associations between dietary intake and Intelligence quotient (IQ). Accordingly, we aimed to examine the association between FI and IQ.

**Study design:** Case-control study.

**Methods:** In this case-control study, 222 girl students aged 9 to 11 years old were randomly chosen from Bandar-Anzali, Iran in 2013. Students with low and moderate IQ were considered as case (n=111) and control (n=111) group, respectively. General and demographic characteristics were collected using interview. United States Department of Agriculture (USDA) household FI questionnaires were also completed. To determine IQ, Wechsler’s revised intelligence test was used.

**Results:** FI was observed in 51% of study participants. The prevalence of FI in case and control group was 58.6% and 22.5%, respectively. The mean IQ was 77.97 ±5.56 in case and 94.6 ±5.47 for control group. It was found that there was an inverse association between FI and low IQ. The results of the multiple variable logistic regression analysis (odds ratio and its 95% confidence interval) showed that, FI 3.46 (1.85, 6.50; *P*>0.001), natural type of delivery 2.45 (1.30, 4.62; *P*=0.006), and father’s low education level 2.97 (1.43, 6.19; *P*=0.004) were the risky factors leading to low IQ.

**Conclusions:** There was an inverse link between FI and IQ. Therefore, it is necessary to pay more attention to FI and its consequences, particularly in mental health of children.

## Introduction

Food and nutrition are fundamental needs in mental and physical health of humans^1^. The range of food insecurity (FI) levels varies from anxiety regarding access to sufficient amounts of food in a household, to severe hunger among children of families who have no food^2^. Job loss, lack of permanent employment, and increasing household dimensions are factors that affect diet, and the age and education level of the household's guardian are effective factors on food insecurity^3^.

Intelligence is one of the most controversial concepts that has been widely defined by many scholars, though one unanimous definition has never been agreed upon which. The reason for this is that intelligence is an abstract concept difficult to define. It is the talent for solving issues or making products, which are valuable in one or more cultures^4^. Since the successful invention of the first Intelligence quotient (IQ) test by Alfred Binet and Theodore Simon in 1905, many researchers have tried to determine those factors effective in measuring the mental abilities of humans. Although there is no doubt regarding the effect of genetic factors, genetics are significantly influenced by factors like nutrition and environment^5,6^. Incomplete and poor nutrition as well as relapsing infection caused by poverty, can stop the brain's growth and evolution and can reduce a child's IQ and learning capacities^7^. Unfortunately, continuing malnutrition in infancy usually leads to permanent mental and physical disabilities^8,9^.

A better understanding of the complex relations between IQ and nutrition, education, and parents' educational, social, and economical levels is necessary in order to design interferences for children's general health^10,11^. Children of poor families have a gradual reduction in their school marks and do not enjoy a good advancement in their education^12^.

Although many studies have been done on FI and also on intelligence in Iran and other countries, only one study has been conducted on the relationship between FI and intelligence on English cases^13^. The results however cannot be generalized to Iranian samples. Various reasons for the spread of FI in Iran’s society as well as different expectations about Iranian infants’ intelligence require that a study be done on this matter. Accordingly, we aimed to verify the association between FI and IQ.

## Methods

### Study Participants

The present study was a case-control, conducted through random sampling in 12 public girls’ primary schools of Bandar Anzali, nortjern Iran during autumn and winter of 2013. The city of Bandar Anzali is the largest and the first northern port of Iran, in the Caspian Sea, 49 degrees and 28 min long, and 37 degrees and 28 degrees latitude, minus the sea level 26 meters. The rainfall in this city is very high, with an average annual rainfall of 1892 mm.

The sampling method was simple random. The aim was to select 91 students with low and poor IQ from three third, fourth, and fifth grades of elementary schools and 91 students with high and average IQ from these three sections. Therefore, in order to find these students according to the criteria in the revised Wechsler intelligence test, before the onset of the case-control study, we did a large cross sectional study with 402 students.

The students were randomly selected based on population density of schools, by age and educational grade. Among 402 students in random and cross-sectional sampling, 138 students in the third grade, 130 students in the fourth grade and 134 students in the fifth grade were enrolled.

Of these, 271 students were placed based on the criteria of Wechsler's revised intelligence scale in case group and 131 students in control group. After placing these students in case and control groups, we had to select for each case, one control, randomly and, if possible, at the same school and in the same class.

To do this, and according to the principles of case-control study, we used soft-ware EPI-info for determining the random numbers in each class. If we did not have controls for the cases in a class, we would ignore all the cases and vice versa.

Finally, out of 402 people, 111 were selected as cases and 111 were taken as controls (a total of 222 cases and controls, instead of 182 people). However, after completing the number of children in the two groups (low and average intelligence), there was no need to continue, but the sampling plan was based on matching each case with the control in the primary school and its level, up to 111 cases and 111 controls (222 samples) continued and was established.

### Ethics statement

Ethical clearance as a master’s thesis was obtained from the Ethics Committee of Tehran University of Medical Sciences (Reg. No. 138). Before the data collection, a written informed consent form was taken from the participants or parents. The study’s objective and procedures regarding the questionnaire and school principles’ intelligence test were explained to the Education Organization of Bandar Anzali, and the students' mothers were invited sign to officially agreements.

### Data Collection

A general questionnaire was used to gather the necessary data regarding the subjects’ general information such as demographic characteristics include maternal and child's age, household size, number of children and socio-economic characteristics including the level of education and occupational status of the guardian and mother of the family, the status of the ownership of the residential home and the living facilities.

The levels of education were classified as illiterate, elementary, cycle, diploma, postgraduate, bachelor, master's, doctoral and the employment status of parents was classified as unemployed, housekeeper, worker, freelancer, administrative employee, managerial staff, but at the time of statistical analysis, the level of literacy was divided into two groups of education, lower than the diploma, diploma and higher than the diploma also the status of employment into two groups of employed and unemployed.

The 18-item USDA questionnaire was used to determine families food security situation; and Wechsler’s revised intelligence test was used to determine the subjects’ intelligence quotients.

Out of all the mothers invited to participate in this study, 12 refused for reasons such as having a patient at home, delivering a baby, or not being able to leave their workplaces. In order to be chosen as the subjects of the study, all students were required to be 9 yr old at the third grade level, 10 yr old at the fourth grade level, and 11 yr old at the fifth grade level. All students were required not to have skipped any grade and to live with their mothers. Finally, all of them were required to have no history of medical illness, such as severe asthma, diabetes, brain tumors, epilepsy, thalassemia major, hypothyroidism, or other similar diseases.

After being examined by a psychologist, students suspected of having hyperactivity disorder, attention deficit (ADHD), depression, or anxiety disorder were omitted from the study after doing the intelligence tests and sampling. Three students suffering from hyperactivity disorder, two students suffering from diabetes type 1, two students suffering from brain tumors, 5 students suffering from severe asthma, and 7 students suffering from epilepsy were identified in the random sampling and omitted from the study groups. Data on parents’ educational and occupational levels, ownership or lack thereof of a private home, household dimensions, number of children, and mothers’ age were gathered from the questionnaire. Furthermore, the mothers were asked regarding the presence of 9 home utilities, including private home and car, washing machine, LCD TV, dishwashing machine, side-by-side refrigerator, handmade carpets, PC/Laptop, and microware. Households having 3 of these items or less were considered to have a poor economy; households with 4 to 6 items were considered average, and those having 7 to 9 items had determined to be on a good economy situation. Each household’s food security was determined based on the answers of the 18-item USDA questionnaire. Households with 0-2 points had food security, those with 3-7 points had FI without hunger, those with 8-12 points had FI with average hunger, and those having more than 13 points had FI with severe hunger^14^.

A score of at least 130 points on the Wechsler’s revised intelligence test was classified as genius, a score from 120 to 129 was classified as clever, a score between 110-119 was classified as high intelligence, a score of 90-109 was classified as average, a score of 80-89 was classified as poor, a score of 70-79 was classified as borderline, and a score of 69 or less was classified as extremely low. The validity of this intelligence test was reported to be 97% for general intelligence, 97% for verbal intelligence and 93% for practical intelligence. The reliability of this test was adjusted in Shiraz University where its coefficient was 88% with academic achievement and 85% with retesting^15^.

Therefore, based on the Wechsler’s scale, students having an IQ from 90-119 were placed in the control group, and those having an IQ from 70-89 were placed in the case group.

The weights of students and their mothers wearing the least amount of clothes and no shoes, were measured by an SECA scale with 0.1 kg accuracy, and their heights without shoes, heels touching the wall, and while facing forward were measured by an SECA stadiometer with 0.1 cm accuracy.

Since subjects' participation in this study was confirmed after their agreements were signed, their family's information was gathered, and the student's IQ were measured, complete confidentiality was maintained, and there were no ethnical problems.

After gathering the required data, SPSS version 16.0 (Chicago, IL, USA) was used to enter and analyze data. To analyze the relationships between variables of quality and quantity and the IQ, single variable regression analysis was done.

Those variables that had a significant association with the IQ were put into a multiple variable logistic regression model, and the final independent variables (after removing interferer variables including age, school and class) were determined using the BackWard method.

## Results

FI among the studied households was 51%, with FI without hunger being 26%, FI with average hunger being 16.5%, and FI with severe hunger being 8.5%, respectively. The association between FI and IQ was completely significant (*P* <0.001).

Less than half of the households in the case group (41.4% of households) had food security status, while in the households of control group; this ratio was 77.5% (Table 1). In addition, FI without hunger, average and severe hunger in households of case group were 30.6%, 21.6% and 6.3%, respectively, while in households of control group were 13.5%, 6.3% % and 2.7%, which had a significant difference (*P* <0.001).

**Table 1:** Absolute and relative abundance and odds ratio of food security status in case and control groups

**Variables**	**Cases**		**Controls**		**OR (95% CI)**	***P *** **value**
	**Number**	**Percent**	**Number**	**Percent**		
Food security	46	41.4	86	77.5	1.00	
Food insecurity without hunger	34	30.6	15	13.5	4.21 (2.10, 8.60)	0.016
Food insecurity with average hunger	24	21.6	7	6.3	6.42 (2.61, 16.00)	0.001
Food insecurity with severe hunger	7	6.4	3	2.7	4.43 (1.11, 17.70)	0.013

As seen in Table 2, those families that had no employed member and the parents' education was less than diploma had a weak economy and FI. The children in these families were more likely to have lower IQ. In families with employed mothers whose children ate breakfast before going to school, the children were more likely to have higher IQ.

**Table 2:** Comparing various factors between students with Wechsler’s scale (more than 90 as controls or less than 90 as cases) using conditional logistic regression (individually matched for age, school and class)

**Variables**	**Case**		**Control**		**OR (95% CI)**	***P*** **value**
	**Number**	**Percent**	**Number**	**Percent**		
Food security status						
Secure	86	77.4	46	41.4	1.00	
Insecure	25	22.5	65	58.5	4.82 (2.71, 8.70)	0.001
Mother's occupation						
Working	15	13.5	29	26.1	1.00	
Not working	96	86.5	82	73.9	3.71 (1.60, 9.81)	0.042
Mother's education						
Below diploma	49	44.1	21	18.9	1.00	
Diploma	50	45.0	51	45.9	0.41 (0.20, 0.82)	0.001
Higher than diploma	12	10.8	39	35.1	0.12 (0.06, 0.32)	0.001
father's education						
Below diploma	47	42.3	14	12.6	1.00	
Diploma	49	44.1	43	38.7	0.33 (0.21, 0.70)	0.001
Higher than diploma	15	13.5	54	48.6	0.11 (0.04, 0.21)	0.001
Family's economy						
Rich	8	7.2	13	11.7	1.00	
Average	42	37.8	55	49.5	1.21 (0.53, 3.32)	0.023
Poor	61	55.0	43	38.7	2.31 (0.91, 6.00)	0.049
Employed member(s) of the family						
None	4	3.6	3	2.7	1.00	
Mother or Father	89	80.2	73	65.8	0.91 (0.44, 4.21)	0.014
Both	18	16.2	35	31.5	0.41 (0.10, 1.93)	0.028
Does the child eat breakfast?						
Yes	40	36.0	71	64.0	1.00	
No	71	64.0	40	36.0	3.22 (1.80, 5.42)	0.001
Type of delivery						
Natural	52	46.9	25	22.5	1.00	
Cesarean	59	53.1	86	77.5	0.33 (0.21, 0.60)	0.001

A child’s IQ had no significant association with mothers' age, BMI, fathers' employment, or owning a private house; household dimensions and number of children (*P* >0.05). The results of the multiple variable logistic regression analysis showed that among all meaningful variables (after individually matching for age, school and class), FI, type of delivery, and father’s low education levels were the independent and risky factors leading to low IQ (Table 3) 

**Table 3:** Risk factors for reducing IQ using multiple conditional logistic regression (individually matched for age, school and class)

**Factors**	**Cases**		**Controls**		**OR (95% CI)** ^a^	***P *** **value**
	**Number**	**Percent**	**Number**	**Percent**		
Food security status						
Secure	86	77.4	46	41.4	1.00	0.001
Insecure	25	22.5	65	58.5	3.46 (1.85, 6.50)	
Father's education						
diploma/ Higher than Diploma	64	57.7	97	87.4	1.00	0.004
Lower than diploma	47	42.3	14	12.6	2.97 (1.43, 6.19)	
Type of delivery						
Cesarean	59	53.1	86	77.5	1.00	0.006
Natural	52	46.9	25	22.5	2.45 (1.30, 4.62)	

^a^ Adjusted for Parent's occupation, education levels, Family's economy, Employed member(s) of the family, Having breakfast and type of delivery

## Discussion

The results of this study showed that 51% of families of primary school children in Bandar Anzali lived in mild to severe FI and the prevalence of FI in case group (low IQ) was 58.6% and in control group (Average IQ) was 22.5%. In addition, food security status, father's educational levels and type of delivery were most related to the children's intelligence.

In Iran, Karam Soltani et al. were the first to investigate the spread of FI (30.5%) among primary schools in Yazd^15^ in a direct study. Similarly, Payab et al.^16^ reported the spread of FI (50.5%) among primary schools of Ray City. In Isfahan, FI was 36.6%^17^, in Asad Abad, Tabriz 36.6%^18^, in Shiraz 44%^19^, in Dezful 37.6%^20^ and in patients with upper GI tract cancers it was 69.2%^21^.

The spread of FI in any place can be influenced by environmental conditions and the occupation of the household's guardian.

A significant association between FI and children's IQ, which was in agreement with the results of Belsky et al. (13), was seen in our study. In other words, children suffering from FI have lower IQ compared with children who have food security. On the one hand, FI destroys the mental and analytical abilities of children by forcing stress on them and increases the possibility of their mental weakness during life. These children's parents are not able to overcome the daily pressures on their children or control their nutrition. An organized and active family environment improves infants' and students' speech, marks, and iIQ^22,23^. Moreover, primary school students need balanced and a variety of foods in order to learn successfully and increase their physical and perceptual activities. FI leads to children's malnutrition and consequently has undesirable and irreparable consequences for their minds and bodies.

The results of this study showed a positive and significant association between mother's employment and child's IQ. Children with a low intelligence level belonged to a family where the mother was a homemaker. Kamkar et al.^24^ and Shamlou and Ghyasvand^25^ also reported a positive and significant relationship between parents' occupation and children's IQ. When parents are employed, they can have more social communication, which can transfer experience to the family. Moreover, a household's income will increase, which means a family's needs can be more easily met. All of these factors can help improve the IQ of children.

The current study found a significant association between father's educational level and child's IQ such that parents whose education was higher than diploma had children that were more intelligent. The results of this study are in agreement with those of Kamkar et al.^24^, Shamluo and Ghyasvan.^25^, Similarly, in Indonesia and the USA a positive and significant relationship between parents' educational level and children's IQ was found^26,27^. The authors reported that, due to their higher awareness and knowledge, educated parents apply more effective points in educating their children. These parents themselves possibly acquired their education because of their high intelligence levels, and thus had more intelligent children through genetic inheritance. Meshkani^28^, however, found no significant relationship between parents' educational level and children's IQ. Meanwhile, people with a higher education are more likely to search for health services and get more health information.

In the present study, there was a significant correlation between IQs and maternal delivery, which is consistent with the study by Khadem et al. They showed that the IQ of children born to cesarean delivery is more than natural delivery^29^.

But in the study by Seidman et al., there was no meaningful relationship between the child's IQ and delivery method^30^. The high IQ in f cesarean delivery than long-term delivery (normal) is probably due to the non-cephalic baby. In normal delivery, due to the presence of birth strokes with headaches and the use of anesthetics and analgesics during this long-term delivery, there will be adverse effects on the brain and fetal skull.

In this study, the adjusted association between household's economic statuses, employed household members, family's ownership of a private house, children's having breakfast and children's IQ was not statistically significant.

The main strength of the current study is the fact that it is the first study ever carried out in Iran to investigate the association between FI and IQ; therefore, this study can be a platform for more studies. One of the limitations of this research is the fact that some parts of the differences in children's IQ can be due to their tiredness, especially when the test was given them after their sports hours.

## Conclusions

IQ had the most significant association with household's food security status, father's educational levels and type of delivery. Since the association between children's IQ with FI was meaningful and inverse (3.4 times more in case families than that in control families), it is necessary to more strongly consider family food security and its consequences, including low IQ.

## Ethical Considerations

Ethical issues (Including plagiarism, informed consent, misconduct, data fabrication and/or falsification, double publication and/or submission, redundancy, etc.) have been completely observed by the authors.

## Acknowledgements

Tehran University of Medical Sciences (Reg No 138) supported this master’s thesis. The researchers of this study would like to extend their heartfelt appreciation to the Research Vice-President of the Health Faculty at Tehran University of Medical Sciences, to the Chancellor of Bandar Anzali Education Organization, to Mrs. Darvishi, the person in charge of Bandar Anzali’s Primary Education, to the principles and headmasters of all primary girl's schools in Bandar Anzali, and finally to all mothers who participated in this study.

## Conflict of interest statement

The authors declare that there is no conflict of interest.

## Funding

None.

## Highlights

- The food insecurity prevalence is notably higher in students with low IQ.
- Father's low level of education is a risk factor for reducing children's IQ.
- Caesarean delivery increases the children's IQ.

## References

[1] Organization WH. Social determinants of mental health. Geneva: WHO; 2014.

[2] Frongillo EA, Nanama S (2006). Development and validation of an experience-based measure of household food insecurity within and across seasons in northern Burkina Faso. J Nutr.

[3] Porter JR, Xie L, Challinor AJ, Cochrane K, Howden SM, Iqbal MM, et al. Food security and food production systems: Cambridge University Press; 2014.

[4] Gardner H, Hatch T (1989). Educational implications of the theory of multiple intelligences. Educational Researcher.

[5] Prado EL, Dewey KG (2014). Nutrition and brain development in early life. Nutr Rev.

[6] Tucker-Drob EM, Briley DA (2014). Continuity of genetic and environmental influences on cognition across the life span: A meta-analysis of longitudinal twin and adoption studies. Psychol Bull.

[7] Barker ED, Kirkham N, Ng J, Jensen SK (2013). Prenatal maternal depression symptoms and nutrition, and child cognitive function. Br J Psychiatry.

[8] Pollit E, Granoff D. Mental and motor development of Peruvian children treated for severe malnutrition. Interam J Psychol 2011; 93-102.

[9] Sudfeld CR, McCoy DC, Fink G, Muhihi A, Bellinger DC, Masanja H (2015). Malnutrition and its determinants are associated with suboptimal cognitive, communication, and motor development in Tanzanian children. J Nutr.

[10] Nyaradi A, Oddy WH, Hickling S, Li J, Foster JK. The relationship between nutrition in infancy and cognitive performance during adolescence. Front Nutr 2015; 1-8. 10.3389/fnut.2015.00002 PMC4451795 26082928

[11] Ronfani L, Brumatti LV, Mariuz M, Tognin V, Bin M, Ferluga V (2015). The complex interaction between home environment, socioeconomic status, maternal IQ and early child neurocognitive development: a multivariate analysis of data collected in a newborn cohort study. PLoS One.

[12] Reiman J, Leighton P. The rich get richer and the poor get prison: Ideology, class, and criminal justice. 10th ed. Routledge; 2015.

[13] Belsky DW, Moffitt TE, Arseneault L, Melchior M, Caspi A (2010). Context and sequelae of food insecurity in children's development. Am J Epidemiol.

[14] Rafiei M, Nord M, Sadeghizadeh A, Entezari MH (2009). Assessing the internal validity of a household survey-based food security measure adapted for use in Iran. J Nutr.

[15] Eshraghian M, Siassi F, Jazayeri G (2007). Obesity and food security in Yazd primary school students. Tehran Univ Med J.

[16] Payab M, Dorosty A, Eshraghian M, Siassi F, Karimi T (2012). Association of food insecurity with some of socioeconomic and nutritional factors in mothers with primary school child in rey city. Iran J Nutr Sci Food Technol.

[17] Mohammadzadeh A, Dorosty A, Eshraghian M (2010). Household food security status and associated factors among high-school students in Esfahan, Iran. Public Health Nutr.

[18] Dastgiri S MS, Totonchi H, Ostadrahimi AR (2006). Influencing Factors on Food Insecurity: A Cross Sectional Study in Tabriz Years 2004-2005. J Ardabil Univ Med Sci.

[19] Ramesh T, Dorosty A, Abdollahi M (2010). Prevalence of food insecurity in household of Shiraz and association with some of socioeconomic and population factors. Iran J Nutr Sci Food Technol.

[20] Hakim S, Dorosty A, Eshraqian M (2011). Relationship between food insecurity and some of socioeconomic factors with BMI among women in Dezfoul. J Sch Public Health Inst Public Health Res.

[21] Daneshi-Maskooni M, Badri-Fariman M, Habibi N, Dorosty-Motlagh A, Yavari H, Kashani A (2017). The Relationship between Food Insecurity and Esophageal and Gastric Cancers: A Case-Control Study. J Res Health Sci.

[22] Vernon PE. Intelligence and Cultural Environment (Psychology Revivals): Routledge; 2014.

[23] Sternberg RJ, Grigorenko EL. Environmental effects on cognitive abilities. Psychology Press; 2014.

[24] Kamkar A ATM, Fararouee M (2001). Assessment of intelligence status and its relationship with educational advance in Yasooj class 5 Primary School Students. Armaghane-danesh.

[25] Shamloo F, Ghiasvand N (1997). Assessment of IQ in Ghazvin class 2 Primary School Students and its association with familial marriage 1996-1997. J Qazvin Univ Med Sci.

[26] Webb K, Horton N, Katz D (2005). Parental IQ and cognitive development of malnourished Indonesian children. Eur J Clin Nutr.

[27] Bliddal M, Olsen J, Støvring H, Eriksen H-LF, Kesmodel US, Sørensen TI (2014). Maternal pre-pregnancy BMI and intelligence quotient (IQ) in 5-year-old children: a cohort based study. PLoS One.

[28] Meshkani Z (1999). Assessment of associated factors to IQ and its relationship with educational advance in secondary School Students. Iran J Child Dis.

[29] Khadem N, Khadivzadeh T (2010). The intelligence quotient of school aged children delivered by cesarean section and vaginal delivery. Iran J Nurs Midwifery Res.

[30] Seidman D, Mashiach S, Laor A, Danon Y, Gale R, Stevenson D (1991). Long-term effects of vacuum and forceps deliveries. Lancet.

